# Conserved structured domains in plant non-coding RNA enod40, their evolution and recruitment of sequences from transposable elements

**DOI:** 10.1093/nargab/lqad091

**Published:** 2023-10-16

**Authors:** Alexander P Gultyaev, Celine Koster, Diederik Cames van Batenburg, Tom Sistermans, Niels van Belle, Daan Vijfvinkel, Andreas Roussis

**Affiliations:** Leiden Institute of Advanced Computer Science, Leiden University, PO Box 9512, 2300 RA Leiden, The Netherlands; Department of Viroscience, Erasmus Medical Center, PO Box 2040, 3000 CA Rotterdam, The Netherlands; Life Science & Technology Honours College, Leiden University, PO Box 9502, 2300 RA Leiden, The Netherlands; Amsterdam University Medical Center, Department of Human Genetics, section Ophthalmogenetics, Location AMC, Meibergdreef 9, Amsterdam, The Netherlands; Leiden Institute of Advanced Computer Science, Leiden University, PO Box 9512, 2300 RA Leiden, The Netherlands; CareRate, Unit E1.165, Stationsplein 45, 3013 AK Rotterdam, The Netherlands; Leiden Institute of Advanced Computer Science, Leiden University, PO Box 9512, 2300 RA Leiden, The Netherlands; Institute of Organismic and Molecular Evolution, Johannes Gutenberg University Mainz, 55128 Mainz, Germany; Leiden Institute of Advanced Computer Science, Leiden University, PO Box 9512, 2300 RA Leiden, The Netherlands; Leiden Institute of Advanced Computer Science, Leiden University, PO Box 9512, 2300 RA Leiden, The Netherlands; National & Kapodistrian University of Athens, Faculty of Biology, Section of Botany, Group Molecular Plant Physiology, Panepistimiopolis - Zografou - Athens, 15784, Greece

## Abstract

Plant long noncoding RNA enod40 is involved in the regulation of symbiotic associations with bacteria, in particular, in nitrogen-fixing root nodules of legumes, and with fungi in phosphate-acquiring arbuscular mycorrhizae formed by various plants. The presence of enod40 genes in plants that do not form such symbioses indicates its other roles in cell physiology. The molecular mechanisms of enod40 RNA function are poorly understood. Enod40 RNAs form several structured domains, conserved to different extents. Due to relatively low sequence similarity, identification of enod40 sequences in plant genomes is not straightforward, and many enod40 genes remain unannotated even in complete genomes. Here, we used comparative structure analysis and sequence similarity searches in order to locate enod40 genes and determine enod40 RNA structures in nitrogen-fixing clade plants and in grasses. The structures combine conserved features with considerable diversity of structural elements, including insertions of structured domain modules originating from transposable elements. Remarkably, these insertions contain sequences similar to tandem repeats and several stem-loops are homologous to microRNA precursors.

## Introduction

Plant enod40 gene has been initially identified as one of the early nodulin genes activated upon the formation of nitrogen-fixing root nodules during the primal stages of symbiotic association of legumes with soil rhizobial bacteria (1,2). It is also involved in the stimulation of colonization of plant roots by fungi with the formation of phosphate-acquiring arbuscular mycorrhizae (3,4). On the other hand, enod40 homologues have been found in multiple non-legume plants, which do not form symbiosis with rhizobial bacteria, including those that do not establish effective mycorrhizal symbioses (5). Identification of enod40 expression in non-symbiotic tissues and studies on its biological effects indicate its importance beyond the regulation of symbiosis (6–12). However, not much is known about the molecular mechanisms of enod40 functions.

In many plant species, enod40 transcripts can be classified as so-called dual RNAs, that is, structured molecules with both RNA-mediated functions and polypeptide-coding capacity (13). Legume enod40 genes contain two conserved regions (coined region I and region II) with short open reading frames (sORFs). Region I codes for a short peptide of 12–13 amino acids, which is also conserved in many, but not all, non-leguminous species, whereas in region II no conserved sORF can be proposed despite the prominent conservation of its core nucleotide sequence (5,8). Translation *in vitro* of enod40 sORFs has been demonstrated and the peptide products have been shown to bind to sucrose synthase (14–16). Such a binding activates sucrose cleavage activity whereas its synthesis activity remains unchanged (17).

The presence of conserved structured domains in enod40 RNAs, even in those lacking conserved sORFs, suggests that the enod40 function is mostly determined by its RNA structure (5). In *Medicago truncatula*, enod40 RNA has been shown to bind a protein MtRBP1 (for *Medicago truncatula* RNA Binding Protein 1) and export it from nuclear speckles into the cytoplasm during nodule development (18). The closest homologs of MtRBP1 in plants are nuclear speckle RNA-binding proteins, regulators of alternative splicing, suggesting that enod40 RNA can regulate the alternative splicing of specific mRNAs upon a switch in organogenesis (13,19,20).

Experimental data on enod40 RNA structure have been obtained for the molecules from only two species, *Glycine max* (21) and *Lupinus luteus* (16). Despite the relatively low sequence similarity, even within legumes, RNA structure probing of these enod40 RNA molecules has demonstrated the formation of homologous structured stem-loop domains, also supported by comparisons with enod40 sequences from other legumes (16,21). RNA structure predictions have identified six stem-loop domains, named domains 1–6 in the 5′-3′ direction, conserved in enod40 RNAs from all legumes or at least in some of them (21). Domains 1–3 are conserved in diverse leguminous species, whereas domain 4 insertion has been observed only in a cluster of plants known to produce indeterminate nodules. Domains 5 and 6 are less conserved; for instance, domain 5 is absent in the *Lupinus luteus* enod40 RNA (16).

Using nucleotide sequence database similarity searches and structure predictions, we have previously identified a number of unannotated enod40 genes and the most conserved structural elements in legume and non-legume enod40 RNAs (5). In particular, a conserved core secondary structure encompassing the region II surrounded by the the lowest part of domain 2 upstream and the domain 3 downstream turned out to be conserved in all enod40 sequences available at that time. Apart from these elements, enod40 RNA structure seems to be highly variable. Although the domain 2 lower closing stem presents a frequently recurring motif GUUUG/CAAAC in both legumes and non-legumes, and the domain itself has a conserved topology with stable extended stem-loop structure, its size varies enormously in a range of 40–200 nucleotides. Domain 1 has not been identified in some non-legume plants, and no conserved structure could be found in non-legumes downstream of domain 3 (5).

Since 2007, no systematic study on the enod40 RNA secondary structure has been published. On the other hand, a number of complete or almost complete plant genomes have been sequenced in this period. Apparently, enod40 gene is conserved in angiosperms. Here, we focused on mining unannotated enod40 genes in the genomic data for two groups of plants: the nitrogen-fixing clade and grasses. The nitrogen-fixing clade comprises the Fabales, Fagales, Cucurbitales and Rosales orders, which contain both species that form nitrogen-fixing root nodule symbiosis with diverse bacteria and those that do not (22,23). Grasses (Poaceae) are an economically important group of plants, with many genomes sequenced and well-studied phylogeny (24). Comparative analysis of predicted RNA structures of retrieved enod40 sequences from genomes of these two clades, presenting the dataset with both closely and distantly related species, refined the main features of enod40 RNA structure and evolution. Surprisingly, the results also showed a striking pattern of non-coding RNA evolution exploiting frequent insertions of transposable elements (TEs) as novel blocks of its functional structure. These insertions share similarities with tandem repeats and microRNA precursors.

## Materials and methods

### Mining of enod40 genes in plant genomes

Enod40 genes were searched for in genomes available in the NCBI Genome database (25) using sequence database similarity search by BLAST program (26). The option ‘BLAST Genomes’ was executed sequentially, using previously identified enod40 sequences as queries for genomes of the most related species according to the phylogenetic trees (24,27–30).

### RNA secondary structure predictions

RNA secondary structures were predicted using the algorithms Fold of RNAstructure Web Servers (31) (https://rna.urmc.rochester.edu/RNAstructureWeb/) and RNAfold of ViennaRNA Web Services (32) (http://rna.tbi.univie.ac.at/#webservices). The calculations were done using temperature 20°C as suitable for enod40 RNAs which are functional in plant roots. Predictions were done separately for specific domains, using the sequences of the domains with putative boundaries derived from alignments to the closely related enod40 genes. As a rule, the lowest free energy conformation was consistent with the typical extended stem-loop structure of a domain, but sometimes one of suboptimal structures was more similar to the consensus.

### Analysis of sequence similarities

Searches for similarities with TEs were carried out using BLAST algorithm (26) with screening corresponding genome from the NCBI Genome database or the REPETDB database of plant transposable elements (33) (https://urgi.versailles.inra.fr/Data/Transposable-elements/REPETDB). In order to detect distantly related sequences, the searches with word size of 7 were used (lower than the usual default 11). For similarities to microRNA genes, BLAST programs in the databases miRBase (34) and PmiREN2.0 (35) were used.

Sequence logos were generated by the WebLogo algorithm (36) (https://weblogo.berkeley.edu/).

Potential peptides encoded by enod40 sequences were identified using the ORF finder program of the NCBI resources (25). Protein motifs in the polypeptides encoded by the ORFs in the putative LINE transposon inserted in the maize enod40-2 were studied using the InterProScan algorithm (37).

## Results

### Searching for enod40 genes

**Figure 1.**
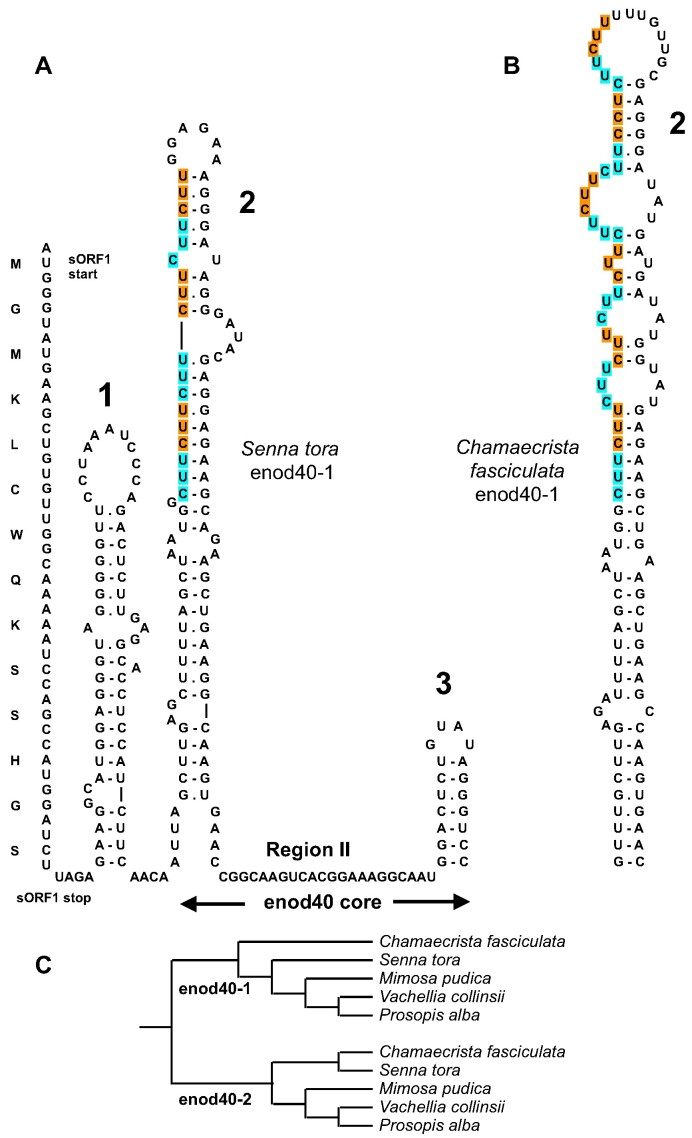
An example of the enod40 RNA structure and its evolution. (**A**) Conserved domains in the *Senna tora* enod40-1 RNA. (**B**) Trinucleotide CUU repeat expansion in the domain 2 of *Chamaecrista fasciculata* enod40-1. (**C**) Evolutionary relationships between two different types of enod40 RNAs in Caesalpinioideae. CUU triplets in the repeat expansion regions are labeled with alternate colours.

The conserved topology of enod40 core structure (Figure 1A) allowed us to localize enod40 genes in almost all available genomes of nitrogen-fixing clade and grasses using a sequential application of BLAST searches followed by RNA secondary structure predictions (see Materials and Methods). The genomic positions of identified enod40 domains are given in the Supplementary Table S1. The fact that enod40 genes were not found in some genomes could be due to incomplete sequencing data.

Similar to previous identification of more than one enod40 gene in some species (9,10,38,39), we identified duplicated enod40 homologues in a number of genomes (Supplementary Table S1). In particular, the majority of legumes have two enod40 genes, with some species comprising three or four copies. On the other hand, in three other orders of the nitrogen-fixing clade (Rosales, Cucurbitales and Fagales) two enod40 genes were found in just a few species. Grasses, typically possess two enod40 genes, although in some species only one or up to four were detected, the latter case being apparently due to minor variations in multiple copies due to polyploidy.

Pairwise comparisons of enod40 genes and phylogenetic clustering showed that some enod40 duplications have occurred before speciation resulting in the extant species. For instance, enod40 genes of the Caesalpinioidae are split into two clusters, with genes from both clusters present in each of the species (Figure 1C). Duplicated enod40 genes of other legumes and grasses (Supplementary Table S1) also exhibited such clustering patterns of two distinct types, consistent with and extending the previous observations (9,10,38).

BLAST searches in available transcriptome assemblies and RNA-seq sequence read archives (SRAs) identified RNA transcripts corresponding to almost all found enod40 homologues (Supplementary Table S1). This supports the expression of enod40 genes, including the duplicated ones. On the other hand, we could not identify RNA sequences of additional enod40 copies in transcriptomic data for some species. Thus, no enod40-2 transcripts of *Juglans* species and related *Pterocarya stenoptera* (Fagales) were identified, while enod40-1 sequences of these plants are covered by RNA-seq SRA reads (Supplementary Table S1). In each of the genomes of *Triticum aestivum* and *Panicum miliaceum* (Poales), we could identify RNA transcripts for all but one enod40 gene out of four and five copies, respectively. Probably, some enod40 gene copies are not expressed or expressed only under specific conditions and/or at specific stages of plant cell development.

### Enod40 sORF1

The conserved short reading frame sORF1 sequence, characterized by the presence of tryptophane in the middle and the histidine-glycine-serine C-terminus (8,40,41), was found in almost all enod40 genes identified in legumes and grasses (Supplementary Table S1). Its typical length varied in the range of 11–13 amino acids. In contrast, enod40 genes in three non-legume orders of the nitrogen-fixing clade are characterized by multiple deviations from or no translatable homologous sORF1 in the species closely related to those possessing it.

**Figure 2.**
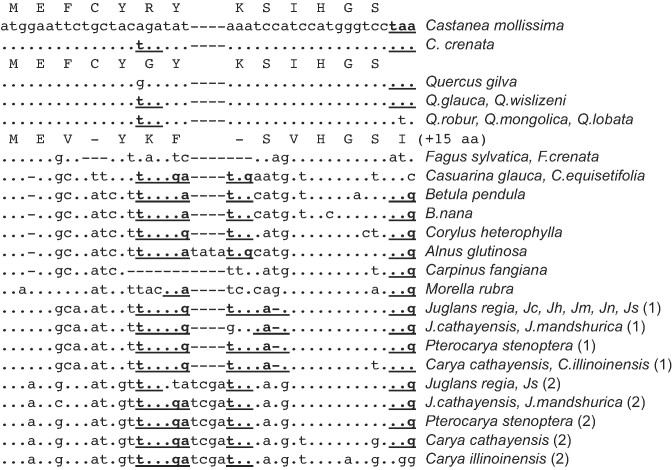
Disruptions of sORF1 reading frame in Fagales species. Dots indicate identical nucleotides. Non-interrupted amino acid sequences are shown. In-frame stop codons are shown in bold and underlined. Jc, *Juglans californica*; Jh, *Juglans hindsii*; Jm, *Juglans microcarpa*; Jn, *Juglans nigra*; Js, *Juglans sigillata*.

In the evolution of Fagales, enod40 apparently has lost its sORF1 coding capacity several times (Figure 2, Supplementary Table S1). For instance, while *Castanea mollissima* and *Quercus gilva* do have typical enod40 sORF1 sequences, both *Castanea* and *Quercus* genera include species with the sORF1 interrupted by a stop-codon. In some *Quercus* species, this nonsense mutation is also accompanied by a mutation disrupting the stop-codon that defines the typical sORF1 C-terminus (Figure 2). In a number of other Fagales genera, the conserved enod40 sORF1 is interrupted by up to three stop-codons, sometimes combined with frameshifts due to indels. Such frameshifts resulting from single nucleotide deletion in the second codon neutralize the effect of the downstream nonsense mutation(s), but, as it has previously been noted for the *Casuarina glauca* enod40 (42), they also inhibit the conserved sORF1 peptide expression. On the other hand, in *Fagus* species the sORF1 is longer because of stop-codon disruption, coding for a peptide with additional 15 amino acids downstream of the conserved histidine-glycine-serine motif (Figure 2). Remarkably, the codons for this motif are also present in Fagales enod40 sequences where they are not translated, suggesting a role in the context of enod40 being a noncoding RNA.

Several Rosales enod40 genes possessed consensus sORF1s, but a number of enod40 RNAs contained sORFs which deviated from the consensus in terms of sequence and/or length, while in some species no obvious sORF1 homologue could be identified (Supplementary Table S1). No sORF1 homologues were found in Cucurbitales, although stop-codons not further than 13 nucleotides upstream of the domain 1 were present in the majority of the species. Maintained codons for the histidine-glycine-serine motif, being apparently not translated because of the in-frame stop-codon upstream, were found only in *Datisca glomerata*.

The maize (*Zea mays*) enod40-2 gene turned out to contain the insertion of 4614 nucleotides between the 8th and 9th codons of its sORF1 (Supplementary Table S1). Sequence analysis of this insertion indicated that this is a long interspersed nuclear element (LINE) with two ORFs, flanked by target site duplication sequences, typical for this type of non-LTR retrotransposons (43). Furthermore, although we could not assign any function to the protein coded by the first ORF, BLAST and protein motif searches showed the second protein to contain endonuclease and reverse transcriptase motifs. Available sequences of transcripts in NR and EST databases, mapped to this enod40-2 gene (accessions EU960460 and DN209550), have no transposon.

### Domain 1

The domain 1 of enod40 RNA structure has previously been identified as a stable stem-loop structure downstream of the sORF1, usually with a purine-rich 5'-half and a pyrimidine-rich 3'-half (14,21,44). It is conserved in legumes, but its localization in the enod40 RNAs of other plants may be less straightforward due to the poor conservation and possible folding of alternative structures (5). For the modelling of domain 1 in enod40 RNAs of the nitrogen-fixing clade and grasses, we used secondary structure predictions of the fragments between sORF1 stop-codon (or homologous position in sORF1-less genes) and domain 2. Relatively stable structures were predicted in all analyzed enod40 RNAs (Supplementary Table S1).

The conserved topology of a single stem-loop with pairings between purine- and pyrimidine-rich halves was predicted in all Fabales, Rosales and Cucurbitales enod40 RNAs. The same shape was conserved in RNAs coded by the enod40-1 genes of Fagales and grasses. However, domains 1 in the enod40-2 molecules of *Carya*, *Pterocarya* and *Juglans* species (Fagales) and of all PACMAD clade plants folded into two stem-loop structures instead of one. Within these clades, such structures were conserved and had optimal or close to optimal values of folding free energy. In some enod40 RNAs of grasses, an additional small hairpin between typical domain 1 structure and domain 2 was predicted. No other conserved alternative structures were predicted.

### Insertion of a transposable element in the domain 1 of *Panicum virgatum*

**Figure 3.**
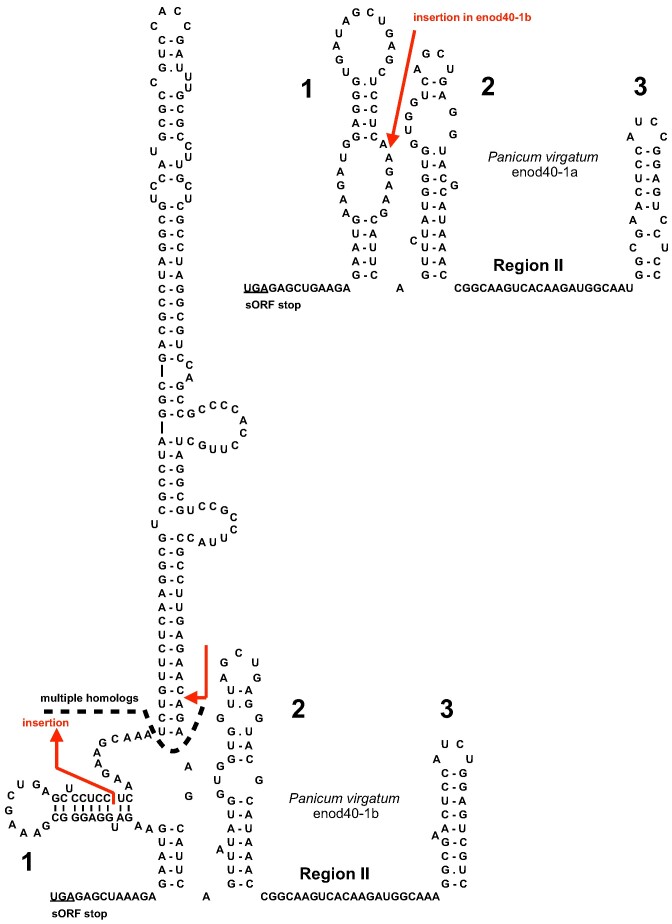
The insertion in the domain 1 of the *Panicum virgatum* enod40-1. The site of insertion in the enod40-1a and the inserted structure in the enod40-1b are indicated by red arrows. The region yielding multiple homologs in BLAST searches is also shown.

Interestingly, two enod40 genes in *Panicum virgatum*, denoted as enod40-1a and enod40-1b, turned out to be close homologues differing by a rather large (138 nucleotides) insertion in domain 1 of enod40-1b. The insertion introduced an extended stable stem-loop structure with a closing stem of 15 bp into the domain (Figure 3). BLAST search in the *P. virgatum* genome using enod40-1b as a query retrieved a large number of high sequence similarity hits with local alignments restricted to the stem-loop insertion: 42 covering more than 90% of the stem-loop, two of them yielding identical sequences. This suggests that enod40-1b insertion is determined by one of the TEs, which are present in multiple dispersed copies in plant genomes (45).

### Domain 2

Domain 2 has previously been identified as an extended stem-loop structure with conserved shape and variable size, located just upstream of the region II (5,21). While the domain size and interior sequences are highly variable, in the majority of enod40 RNA from both legume and non-legume plants the domain 2 has been predicted with a conserved closing double-helical stem GUUUG/CAAAC or its variations.

Comparison of domains 2 in enod40 genes, mined in genomic sequences (Supplementary Table S1), showed that the domain size and sequence variations in the plants of nitrogen-fixing clade were sometimes determined by insertions of trinucleotide repeats. For instance, in enod40-1 RNAs of the two closely related *Senna tora* and *Chamaecrista fasciculata*, a trinucleotide repeat expansion occurred, with 6 and 12 CUU triplets, respectively (Figure 1). Trinucleotide repeat expansions were also found in *Chamaecrista fasciculata* enod40-2 (4 UGA triplets paired to 4 UUA), *Morus alba* (5 GAA vs. 3 GAA in related *Artocarpus camansi*) and *Quercus* species (4 GAA vs. 3 GAA in related *Castanea*). In the domains 2 of enod40 RNAs from grasses, more than two triplet repeats were only found in the enod40-1 of *Panicum virgatum* (3 GGU).

**Figure 4.**
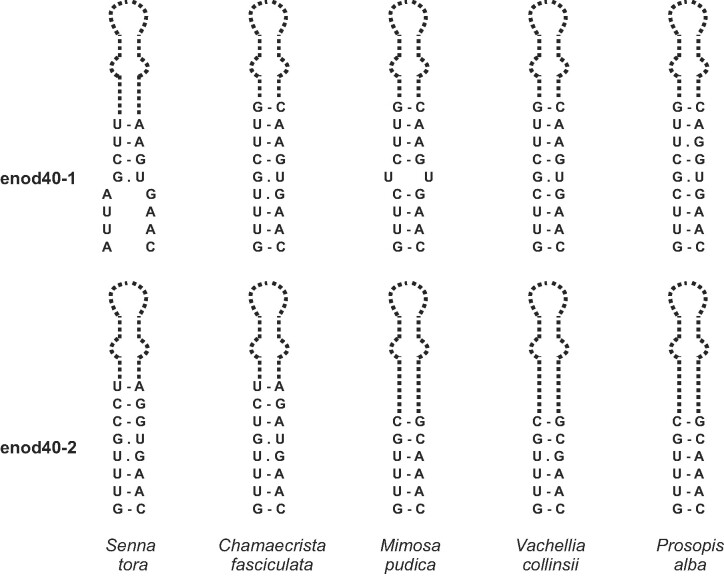
Variation in the closing stems of domains 2 of enod40 RNAs from the Caesalpinioideae species. The domain interiors are shown only schematically, and not in scale.

**Figure 5.**
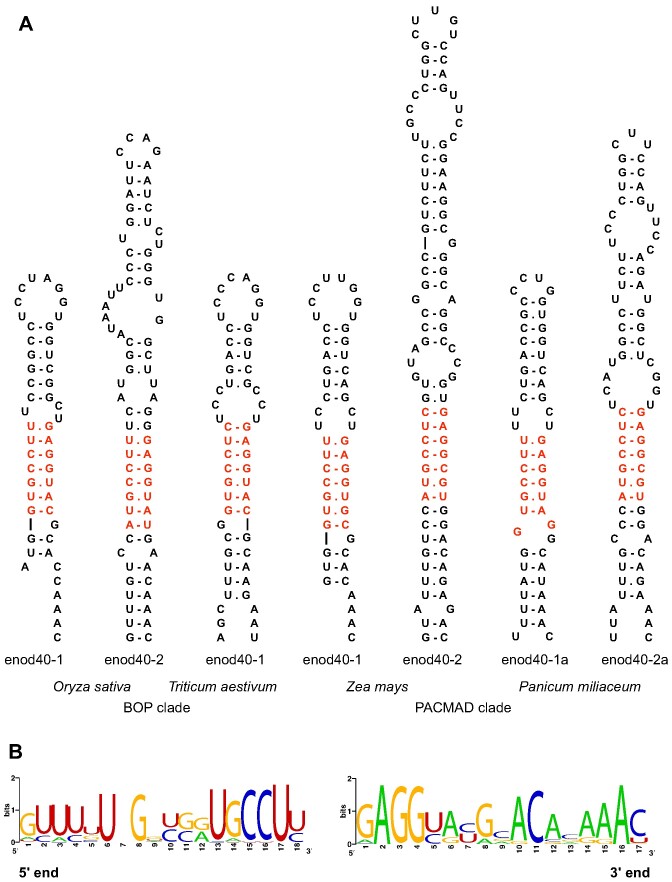
Variations in the domain 2 structures of enod40 RNAs of grasses. (**A**) Examples of structures. The most conserved part is in red. (**B**) Sequence logo's of the domain termini.

As far as the closing stem of domain 2 is concerned, enod40 RNAs with possible pairing between the CAAAC sequence or its homologues at the 5'end of the region II and the 5'proximal nucleotides of the domain 2 were found in the majority of species (Figure 4). However, in some enod40 RNA structures considerable deviations from the GUUUG/CAAAC consensus disrupted this stem. In particular, such disruptions are typical in enod40 RNAs of grasses, that is compensated by evolutionary stabilization of enclosed pairings with the consensus RUGCCUY/GAGGYAY, where R is G or A and Y is C or U (Figure 5). Despite variability of the closing stem and interior part of domain 2, no alternative structures were predicted in its region.

### Insertions of TEs in domain 2

**Figure 6.**
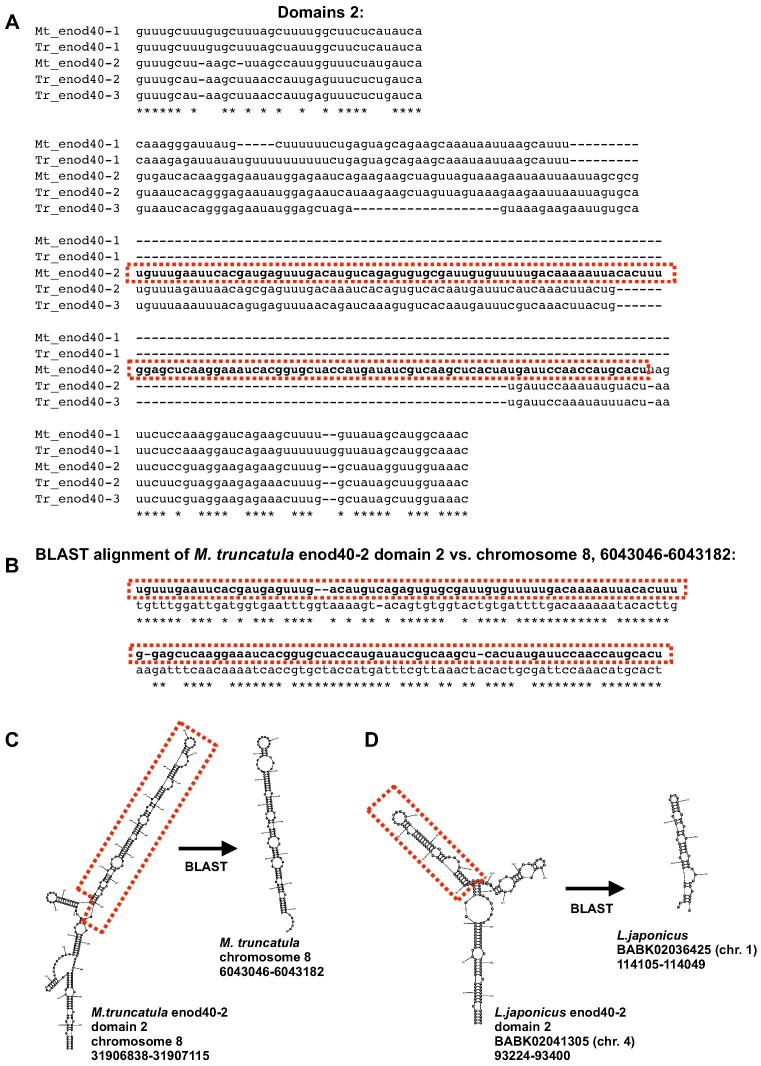
Insertions in the enod40 RNAs of *M. truncatula* and *L. japonicus*. (**A**) Alignment of related *M.truncatula* and *T.repens* enod40 sequences. (**B**) One of the multiple BLAST alignments corresponding to the insertion in the *M. truncatula* enod40-2. (**C**, **D**) Structures of the *M. truncatula* and *L. japonicus* enod40-2 domains 2 and their homologs retrieved by BLAST. The enod40 sequences and structures that have multiple homologs are framed.

In several legume enod40 RNAs domains 2 contain insertions originating from putative DNA transpositions. Thus, BLAST search in the *Medicago truncatula* genome with its enod40-2 domain 2 as the query yields 15 significant (*E* ≤ 2e-06) hits with the lengths of about 130 nucleotides that cover at least 90% of the insertion in the domain as compared to its close homologues (Figure 6A, B). These homologous sequences can form extended stem-loop structures, like in the enod40-2 domain 2 (Figure 6C). The insertion occurred before speciation of *M. truncatula* and *Trifolium repens*, because two of three *T. repens* enod40 genes contain its parts (Figure 6A). Moreover, blast search for sequences similar to the *M. truncatula* enod40-2 domain 2 in the *T. repens* genome yields even more significant alignments (45) than in the *M. truncatula* that cover at least 90% of the insertion. It should be noted that diversity of these sequences is larger, as some alignments depict higher *E*-values, up to 0.008.

The domain 2 of the *Lotus japonicus* enod40-2 also contains a similar type of insertion. One of the arms in the predicted Y-shaped structure of this domain (21) was found to have a number of homologous sequences in the *L. japonicus* genome that yield 35 BLAST alignments with >90% coverage and *E*-value at most 0.008. Not surprisingly, these fragments can form similar stem-loop structures (Figure 6D).

### Region II variations

Previously, the consensus CGGCAAGUCA-N(6)-GGCAAN sequence has been suggested for the region II core located between domains 2 and 3 (5,41). The majority of enod40 RNAs of both nitrogen-fixing clade species and grasses did satisfy this pattern. Typical deviations were insertions of up to three nucleotides into the spacer between two conserved sequences or just downstream of the second GGC motif and deletions of one or two adenines at the latter location. Thus the consensus CGGCAAGUCA-N(6,9)-GGC turned out to be conserved in all but two cases: substitution of the 5'terminal C by U in the *Oryza meyeriana* enod40-1 and deletion of the second GGC motif together with the half of the spacer in *Carya* species. A unique deletion of the half of the region II together with the domain 3 occurred in the *Lupinus albus* enod40-2, leaving just the CGGCAAGUC part, in contrast to the enod40-2 homologues from *L. angustifolius* (Supplementary Table S1) and *L. luteus*, mRNA accession AF352374 (16), which have no deviations from the consensus.

### Domain 3

**Figure 7.**
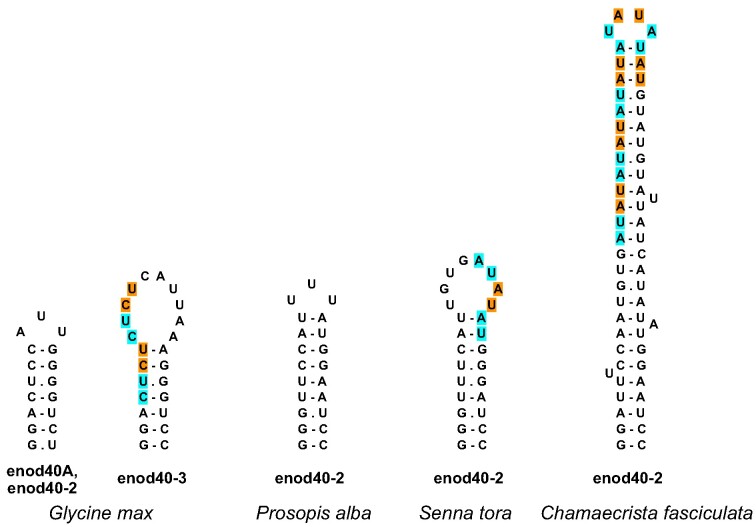
Examples of domain 3 variation in legume enod40 RNAs. Dinucleotide repeat expansions are labeled with alternate colours.

Domain 3 is the most conserved enod40 structure, a relatively small, yet stable, hairpin (5,21), predicted without alternative suboptimal structures. In some enod40 genes of the nitrogen-fixing clade we found more extended hairpins determined by expansions of dinucleotide repeats, for instance, four CU repeats in the *Glycine max* enod40-3 and *Macrotyloma uniflorum* enod40-2, five AU in the *Arachis* enod40-3 and enod40-4, 3 AU in the *Senna tora* enod40-2, 10 AU in the *Chamaecrista fasciculata* enod40-2, 3 AU in *Fagus sylvatica* (Figure 7). Similar to domain 2, domain 3 is characterized by more conserved closing stem and variable apical part.

### Domains 4 in leguminous enod40 genes are derived from TEs

Homologous extended stem-loop structures of domain 4 have previously been identified in a group of closely related leguminous plants known to produce indeterminate nodules, such as *Medicago truncatula, Trifolium repens, Pisum sativum and Vicia sativa* (21). The structures are folded by sequences inserted in the enod40 RNAs of this group in the regions between the more conserved domains 3 and 5.

**Figure 8.**
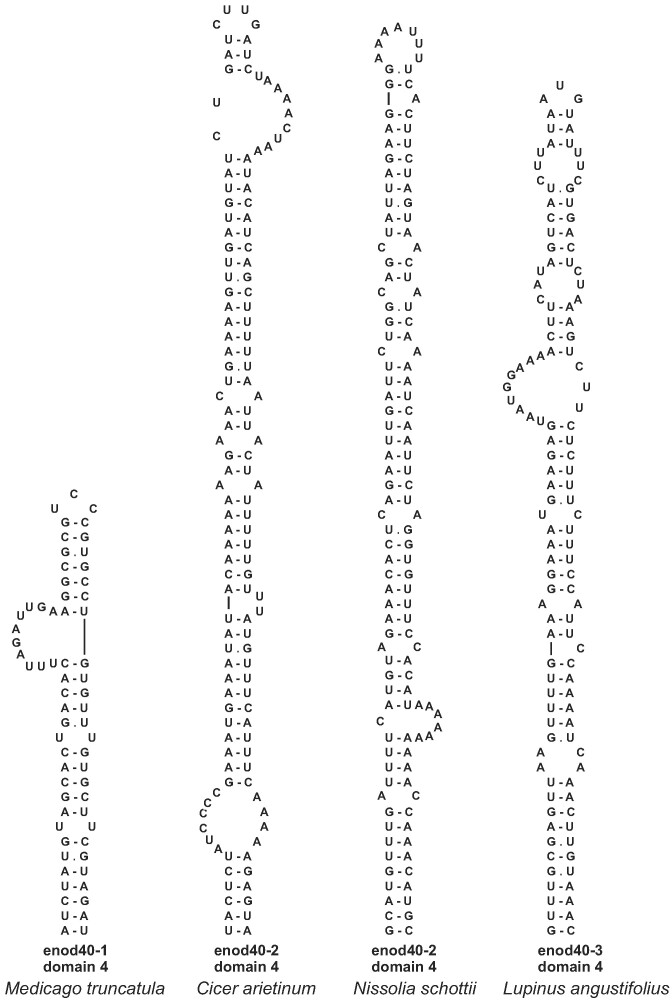
Domains 4 in legume enod40 RNAs. The *M. truncatula* enod40-1 domain 4 structure has previously been suggested along with its homologues of the Medicago/Trifolium clade (21).

Apart from this clade, we found extended stem-loop structures at positions corresponding to domain 4 only in a few enod40 RNAs mined in legume genomes, namely, in the *Cicer arietinum* enod40-2, *Nissolia schottii* enod40-2 and the enod40-3 of two *Lupinus* species (Figure 8, Supplementary Table S1). These structures most likely originated from diverse DNA transpositions, because BLAST searches in corresponding genomes returned multiple similar sequences. Thus, BLAST search in the *C. arietinum* genome with the *C. arietinum* enod40-2 yielded 108 significant (*E* < 1e-03) hits corresponding to its domain 4, seven of which covered each more than 90% of the domain sequence. In the *N.schottii* genome, 38 fragments with coverage of more than 90% of the *N.schottii* enod40-2 domain 4 were found among significant BLAST alignments, with many more hits corresponding to smaller parts of the domain. In the *L. angustifolius* genome, 27 fragments with significant BLAST alignments each covering more than 90% of the *L. angustifolius* enod40-3 domain 4 were found on the chromosome LG17, where the gene is located, and such hits were present on all 20 chromosomes. Domain 4 of the *L. albus* enod40-3 is very similar to its *L. angustifolius* homologue and therefore has the same origin.

Next, we searched for sequences similar to the domains 4 of enod40 RNAs from the Medicago/Trifolium clade in order to check whether these domains could also be derived from DNA transpositions. Indeed, many significant (*E* < 0.001) BLAST hits corresponding to the domains 4 of *M. truncatula, T. repens, P. sativum* and *V. sativa* enod40 RNAs were found in all four genomes. With any of the four domain 4 - containing enod40 sequences of these species (Supplementary Table S1) as the BLAST queries, the searches in genomes of *M. truncatula* and *T. repens* yielded more hits than those in *P. sativum* and *V. sativa*. The domain 4 of *T. repens* enod40-1 produced the largest number of significant alignments: 172, 67, 15 and 10 in genomes of *T. repens*, *M. truncatula*, *V. sativa* and *P. sativum*, respectively. Out of these 264 alignments, 12 covered more than 90% of the domain sequence: 6, 4 and 2 in genomes of *T. repens*, *M. truncatula* and *V. sativa*, respectively. Among 60 significant BLAST alignments to M.truncatula enod40-1 sequence, only one covered more than 90% of its domain 4, in *M. truncatula* genome, involving the same sequence as given by one of the best hits with the *T. repens* enod40-1 query. Searching in the *T. repens* genome with the *P. sativum* enod40 query also yielded one such hit, which was not retrieved with other queries. On the other hand, it should be noted that the majority of significant BLAST alignments covered only the 5' half of the enod40 domains 4.

### Clade-specific enod40 RNA structural elements

Stem-loop structures of domain 5 and 6 have previously been identified by structure probing of the G. max enod40 RNA and shown to be conserved in a number of leguminous plants (21). Homologous domains have not been found in non-leguminous species (5), and the absence of domain 5 in the legume *Lupinus luteus* enod40 RNA has been shown by structure probing (16).

Identification of enod40 genes in the broader phylogenetic range allowed us to determine the extent of conservation of domains 5 and 6 more precisely. It turned out that these domains could be found only in the Papilionoideae subfamily of Leguminosae (27). Within this subfamily, conserved domain 6 can fold in at least one enod40 RNA of all species (Supplementary Table 1). Domain 5 is less conserved, it is absent in a number of enod40 RNAs with stable domain 6 homologues, and in several Papilionoideae plants it was not found in any of the identified enod40 RNAs.

Of course, the absence of the homologous stem-loops does not mean the absence of secondary structure, as alternative structures may be folded as well. However, without experimental evidence or phylogenetic support it is difficult to verify the existence and functional relevance of alternatives.

Notably, a stable stem-loop structure upstream of the sORF region was predicted in enod40 RNAs of several Rosales species (Supplementary Figure S1). In particular, some enod40 RNAs formed the hairpin with perfect double-helical stem consisting of 22 bp. The location of this hairpin just upstream of the sORF1 start codon suggests its possible involvement in translational regulation, but no correlation was found between its folding and the presence of translated consensus sORF1 peptide.

### Similarities of enod40 domain insertions to repetitive elements

Sequence database similarity searches in the RepetDB database of plant transposable elements (33) yielded several BLAST alignments between insertions into the enod40 structures and the database entries. The most significant (*E*-values in the range between 1e-05 and 5e-05) similarities were revealed between domains 4 of the enod40 RNAs from the Medicago/Trifolium clade and the *M. truncatula* transposon ‘Mtru_TEdenovoGr-B-G6045-Map3’, classified by the database as a class I LTR retrotransposon of the Ty1/Copia type. While this transposon, denoted here as Mtru_G6045, contains only parts of the functional full-length LTR retrotransposon, TBLASTN alignments showed its close similarity to the Mtr30 family of *M. truncatula* LTR retrotransposons (46). The Mtru_G6045 region that shares similarity with the *M. truncatula* enod40 domain 4 is located in the middle of the transposon, downstream of the region containing codons corresponding to the part between integrase and revertase domains of the transposase (Supplementary Figure S2A). Remarkably, the similarity to enod40 involves the sequence which is duplicated with few deviations at the distance of 27 nucleotides.

Inspection of the results of BLAST searches in genomic sequences showed that other enod40 domain insertions shared similarities with fragments of repetitive motifs as well (Supplementary Figures S2, S3). The arrangements of these motifs resemble that of tandem repeats, which are abundant in eukaryotic genomes, plants included (reviewed by e.g. (47). The repeating units (monomers) sharing similarities with enod40 domains are either adjacent or separated by short spacers. With the exception of the *C. arietinum* tandem repeat monomer of more 2000 nucleotides, the monomers are in the range of 60–210 nucleotides.

Two types of structural context of enod40 RNA similarity regions to the tandem repeats could be distinguished. In some enod40 RNAs these sequences approximately correspond to either 5' or 3' half of the inserted stem-loop structure (Supplementary Figure S2), while in others the similarity includes complete stem-loop (Supplementary Figure S3). Although the BLAST alignment of *C. arietinum* enod40-2 domain 4 to tandem repeat covering only the apical part of the domain (Supplementary Figure S3D) seems to present an exception, the optimal local alignment calculated by the Smith-Waterman algorithm of the EMBOSS Water program (48) extends this similarity, covering the whole domain (not shown).

Both *M. truncatula* enod40-2 domain 2 right arm insertion and homologous repeated motif (Supplementary Figure S2B) share significant similarity with the RepetDB database entry Mtru_TEdenovoGr-B-G7965-Map3, denoted here as Mtru_G7965, revealed by the database BLAST searches with E-values of 2e-04 and 1e-05, respectively. In four full-length G7965 copies, which were identified in the *M. truncatula* genome, this similarity encompassed just a part of single monomer of the repeat that also shared similarity with the enod40-2 domain 2 (Supplementary Figure S2B). Blast search in the genome showed the tandem repeat multiplication of four monomers to be unique, while multiple hits mapped either to this part or to the remaining downstream region of the monomer. It should be noted that the Mtru_G7965 repetitive element has not been classified by the RepetDB database (33) and probably is not a TE.

BLAST searches showed that in the *P. virgatum* genome the fragment of the chromosome 5K of 709 nucleotides (complement positions 13101713–13101005), bearing the second monomer of the tandem repeat at positions 13101889–13101535 (Supplementary Figure S2C), has multiple imperfect full-length copies on different chromosomes. However, the tandem repeat on chromosome 5K is unique. Both the 5'-proximal nucleotides of its monomer and similar sequence in the enod40-1b domain 1 insertion contain 2.5 repeats of palindrome AGGCG(T/A)CGCCT (Supplementary Figure S2C). BLAST searches in the RepetDB database with the 709 nucleotides - long repetitive sequence and enod40 insertion yielded alignments with *E*-values of 2e-05 and 0.001, respectively, to an unclassified repetitive DNA element ‘Sbic_TEdenovoGr-B-G2377-Map4’ of closely related species *Sorghum bicolor*. These alignments covered just the region of palindromic repeats.

Some of the tandem repeats related to the enod40 insertions overlap with the protein-coding genes. Thus, the 125–127 nucleotides—long repeats of the *L. angustifolius* (Supplementary Figure S3C) are located in the intron of a gene for a probable polygalacturonase (GeneID 109351179). Interestingly, a large part of this gene, including the repeat-containing intron and flanking exons, is in turn duplicated, leading to a tandem repeat with monomer length of 1398 nucleotides. The second monomer of the *C. arietinum* tandem repeat region (Supplementary Figure S3D) overlaps with the first exon and part of the first intron of the GDSL esterase/lipase gene (GeneID 101500147).

### Similarities of enod40 domain insertions to microRNA stem-loops

As the extended stem-loops of enod40 RNA domains resemble the structures of microRNA precursors (reviewed by e.g. 49), sequence similarities were searched for in the microRNA databases. Indeed, BLAST searches in the miRBase (34) identified significant similarities of *M. truncatula* enod40-1 domain 4, enod40-2 domain 2 insertion and *C. arietinum* enod40-2 domain 4 to mtr-MIR 2644, mtr-MIR169d and mtr-MIR5281d precursors, respectively, with E-values between 3e-11 and 4e-04. The mtr-MIR2644 similarity to the *M. truncatula* enod40-1 domain 4 was also confirmed by the search in the PmiREN database of plant microRNAs (35).

Although the sequences of these microRNA genes are not among the best hits in genomic BLAST searches with the same enod40 queries, with rather high E-values, similar structural contexts of aligned fragments suggest that the detected mapping of nucleotides does reflect the homology of microRNA precursor and enod40 domain structures (Supplementary Figures S4–S6). The sequence homology between the *M. truncatula* enod40-1 domain 4 and the mtr-MIR2644 (Supllementary Figure S4) involves the fragment that yields multiple BLAST hits in *M. truncatula* genome, including the tandem repeats (Supplementary Figure S1). In both enod40 domain 4 and mtr-MIR2644 precursor structures these sequences correspond to approximately halves of the domains consisting of helices and internal loops. The stem-loop insertion in the *M.truncatula* enod40-2 domain 2 is homologous to the sequence complementary to the stem-loop in the mtr-MIR169d, which in turn is an insertion as compared to other MIR169 isoforms such as mtr-MIR169c (Supplementary Figure S5). Homologous sequences in the *C. arietinum* enod40-2 domain 4 and mtr-MIR5281d occupy homologous positions in the apical parts of the structures (Supplementary Figure S6).

BLAST searches in the sequence read archives (SRAs) containing small RNA libraries of corresponding species identified reads that indicated to possible microRNA-like processing of enod40 domain insertions. Thus, the recurrent presence of reads that mapped to the double-stranded regions of *M. truncatula* enod40-1 domain 4 and enod40-2 domain 2 (Supplementary Figures S4, S5) was detected in several small RNA libraries, e.g. with SRA accessions SRX651005, SRX651006, SRX087134. Small RNA library reads that mapped to certain parts of enod40 domain insertions in *C. arietinum* (e.g. SRA accession SRX19984047), *Lotus japonicus* (e.g. SRX1613597), *Lupinus angustifolius* (e.g. SRX2635185) and *Panicumvirgatum* (e.g. SRX3837804) were also repeatedly found, albeit with relatively low abundance. Although a number of such reads mapped to the stems of inserted structures, some contained the nucleotides of apical loops, unlikely to correspond to small RNAs produced by microRNA processing machinery (49).

### Similarities of enod40 domain insertions to MITEs

Relatively weak similarities between the enod40 domains and microRNA precursors suggest that these structures are distant relatives with common ancestors originating from TE sequences. The shapes of both enod40 RNA insertions and microRNA stem-loops are similar to those of miniature inverted-repeat transposable elements (MITEs), which are present in high copy number in plants (45,50). A number of the TE-derived microRNA precursors originated from MITE structures indeed (51–53). In particular, the mtr-MIR169d and mtr-MIR5281d stem-loops, which share similarities with the *M. truncatula* enod40-2 domain 2 insertion and *C. arietinum* enod40-2 domain 4, respectively (Supplementary Figures S5, S6), have been suggested to be related to MITE superfamilies PIF/Harbinger and Tc1/Mariner, respectively (53). The BLAST alignments of these enod40 insertions versus the representative copies of microRNA-related MITEs turned out to cover approximately the same homologous regions as identified in the corresponding enod40/microRNA comparisons (Supplementary Figures S5–S7A,B). Notably, the sizes of the inserted stem-loops are close to those of their putative MITE homologs, and sequence similarities extend to the (parts of) terminal inverted repeats (TIRs) (Supplementary Figure S7A, B).

Comparisons of other enod40 insertions to the known MITE families (reviewed by (54,55) revealed only a weak similarity between *N. schottii* enod40-2 domain 4 and the AhMITE1 family sequences from closely related species *Arachis hypogaea* (56,57), with BLAST alignments covering only small parts of stem-loop sequences (Supplementary Figure S7C). On the other hand, inspection of sequences flanking the BLAST hits of this domain in the *N. schottii* genome revealed the regions exhibiting typical MITE features (45): extended TIRs flanked by target site duplications (TSDs). In turn, using one of these regions as the BLAST query retrieved more copies of the N. schottii MITE-like stem-loops homologous to the enod40-2 domain 4 (Supplementary Figure S7C).

Typical MITE features were also detected in the *P. virgatum* genome regions highly similar to the enod40-1b domain 1 insertion. Putative MITE-like stem-loops identical or deviating by only one substitution from the domain stem-loop turned out to be flanked by TSDs of the same length of 8 nucleotides with different duplicated sequences (Supplementary Figure S7D). Interestingly, this TSD pattern is absent in the enod40-1b domain 1.

## Discussion

The secondary structures of enod40 RNAs, identified in this work, demonstrate a remarkable combination of conserved features with high diversity of structural elements. In addition to its main core, which consists of the conserved region II and adjacent stems of domains 2 and 3 (5), the enod40 evolution has been creating other less conserved structures that could contribute to the enod40 functions.

Various evolutionary processes produced diverse enod40 RNA structures. To a certain extent, the diversity of domain 2 sizes resulted from frequent trinucleotide repeat expansions with subsequent restoration of the rod-like shape (Figure 1) or insertions of stem-loops derived from DNA repeats and mobile elements (Figure 6). Furthermore, stem-loop insertions with sequences related to repetitive DNA seem to be actively exploited as novel domains 4 in some of enod40 RNAs of leguminous plants (Figure 8). Such repeated insertions at approximately the same position downstream of the conserved domain 3 are clearly non-random. It could be noted that repetitive sequences can also trigger complex changes in gene regulation such as paramutation (58).

Recruitment of sequences derived from TEs as functional domains in non-coding RNAs has previously been revealed (59–63). The best studied are the repeat domains of the eutherian long noncoding RNA Xist, which have originated from various TEs (59). While the Xist evolution involved multiplication of tandem repeat monomers with various copy numbers in different species, the enod40 insertions identified in this work just picked up single motifs of mobile elements that participated in tandem repeats elsewhere.

Acquisition of structural motifs recognized by proteins has been suggested to be the main reason for systematic integration of sequences derived from TEs into noncoding RNAs (61,62,64,65). It is also likely the case for insertions in enod40 RNA, as protein binding is essential in enod40 functions (13,18). Another, not mutually exclusive reason, related to enod40-induced relocalization of bound proteins (13,18) is modulation of its localization, observed for a number of other ncRNAs upon acquisition of TE-derived sequences (66,67).

In the enod40 gene, repeated insertions of TE-derived sequences at similar locations occur in a way that minimally disturb the encoded RNA structure. Such insertions of new modules may create an opportunity to adapt it rather than to destroy its function. None of the insertions of new stem–loops has led to disappearance of more conserved structural elements in enod40 RNA. Moreover, structural similarities between the additional stem-loops acquired independently from unrelated repetitive elements suggests that these domains underwent an adaptation that contributed to enod40 functioning. Presumably, enod40 RNAs have not acquired them directly from tandem repeats, because the tandem repeat monomers are not among the best BLAST hits of enod40 queries. Sequences similar to the entire or almost entire stem-loop insertions, also able to fold into similar structures, can be found elsewhere in genomes even in cases when only a half of the stem-loop shares similarity with a tandem repeat monomer. It seems that these monomeric sequences, dispersed in genome by TEs, can both carry out some function in the stem-loop structural context and undergo tandem repeat expansion. The enod40 stem-loops get this function, with a proper structural context probably gained before insertion into enod40 genes, but tandem repeat expansions of related sequences occurred only at other genomic locations.

Sequence comparisons showed that enod40 stem-loop insertions could originate from diverse TE types, like e.g. LTR-retrotransposons and MITEs. The same has been shown for structurally similar microRNA precursors (51). Insertions of MITE stem-loops into enod40 genes can provide pre-formed structures which may undergo further adaptation for enod40 functions. In cases of enod40 domain insertions with multiple homologous stem-loops with no visible resemblance to MITEs, like e.g. *Lupinus* enod40-3 domain 4, MITE origins are still plausible. It should be noted that plant genomes contain many TE-derived inverted repeat insertions that may not be classified as MITEs because MITE components have been deleted after insertion (68).

Intriguingly, microRNAs processed from precursors that are similar to enod40 insertions have been shown to be involved in the regulation of plant symbiosis with bacteria and fungi. Thus, mtr-MIR169 isoforms are differentially expressed in nodules (69,70), mtr-MIR169d and mtr-MIR5281 in mycorrhizal roots (71), and the PmiREN database (35) reports the mtr-MIR2644 star microRNA (mtr-MIR2644-5p) expression in nodules. Furthermore, the soybean MIR169c isoform, which does not contain the insertion that is shared by the *Medicago* MIR169d precursor and enod40-2 domain 2 (Supplementary Figure S5), regulates the enod40 gene expression by regulating the level of a transcriptional factor GmNFYA-C (72). It is tempting to speculate that insertions of microRNA-like stem-loops into the enod40 RNA structure might modulate the correlations between enod40 and microRNA interactions with other molecules in the control of plant symbiosis signalling.

The recurrent presence of specific reads in small RNA libraries suggests a possibility of microRNA-like processing of some enod40 stem-loops, although it may be less efficient than the cleavage of canonical microRNA precursors. Furthermore, many such reads were longer than 22 nucleotides and therefore unlikely to correspond to bona fide microRNAs (73).

Apparently, the fuctioning of enod40 RNA is mainly determined by its conserved core that consists of the region II and adjacent closing stems of domains 2 and 3. This core is likely to determine enod40 RNA localization signal and/or affinity to proteins, which can be modified by more variable enod40 parts. In the enod40 RNA binding and relocation of proteins belonging to the family of splicing modulators (20), various combinations of stem-loops may determine diverse sets of bound proteins and lead to different patterns of alternative splicing of multiple transcripts. Future studies could elucidate the roles of structured enod40 RNA domains in plant cell physiology.

## Supplementary Material

lqad091_Supplemental_Files

## Data Availability

Sequence related information presented in this study (Data S1) is available on FigShare at https://doi.org/10.6084/m9.figshare.24173826 to ensure reproducibility. Additional supplementary material is available on the journal website.
