# Supplementary material for: Conserved structured domains in plant non-coding RNA enod40, their evolution and recruitment of sequences from transposable elements
Source: NAR Genom Bioinform. 2023 Oct 16;5(4):lqad091. doi: 10.1093/nargab/lqad091 (PMC10578108; doi:10.1093/nargab/lqad091)
Supplement: lqad091_Supplemental_Files [file lqad091_supplemental_files.zip › Supplementary_Figures_enod40_v6.pdf]

Supplementary Figures

for the manuscript by Gultyaev AP et al.

"Conserved structured domains in plant non-coding RNA enod40, their evolution and recruitment of sequences from transposable elements"

A

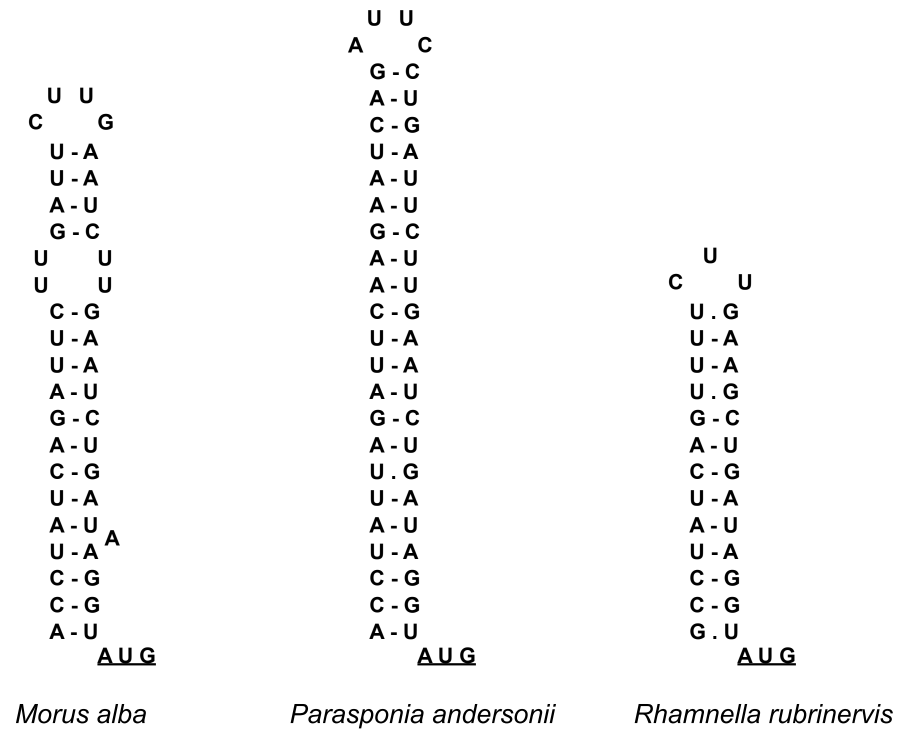

B

```
                                                                                 sORF1
                                                                                 start
aacctatcagattcttgattc------ttgaatcttgaatctgata--aggt---atgg Morus alba
aacatatcagattcttgattc------ttgattcttgaatctgatttcaggcc--atgg Artocarpus camansi enod40-1
aacctatcagattcttgattc------ttgattcttgattctgatt--aggcc--atgg Artocarpus camansi enod40-2
aacttatcagattcttgattc------tttatccttgaatctgat---agggatatgg Ficus carica
aacctattagattcaagaatcagattcctgattcttgaatctgat---aggt---atgg Parasponia andersonii
agcctatcagattcaagaatcagattcctgattcttgaatctgat---aggt---atgg Trema orientale
aaccaatcagattcaagaataatattcttgattcttgaatctgat---aggt---atgg Humulus lupulus
aacctatcagattcaagaatcagatctttgattcttgaatctgat---aggt---atgg Cannabis sativa
agcctatcagtttt-------------------cttgaatctgat---aggaaa-atgg Ziziphus jujuba
agcctatcagattt------------------tcttgaatctgat---aggaaa-atgg Ochetophila trinervis
agcctatcagtttt-------------------cttgaagctgat---aggt---atgg Rhamnella rubrinervis
```

Supplementary Figure S1. Predicted domains S in the enod40 RNAs of Rosales species. (A) Examples of stem-loop structures of different sizes. (B) Structure-guided alignment of domain S sequences. Paired regions in the alignment are labeled in orange. The sORF1 start-codons are in bold and underlined.

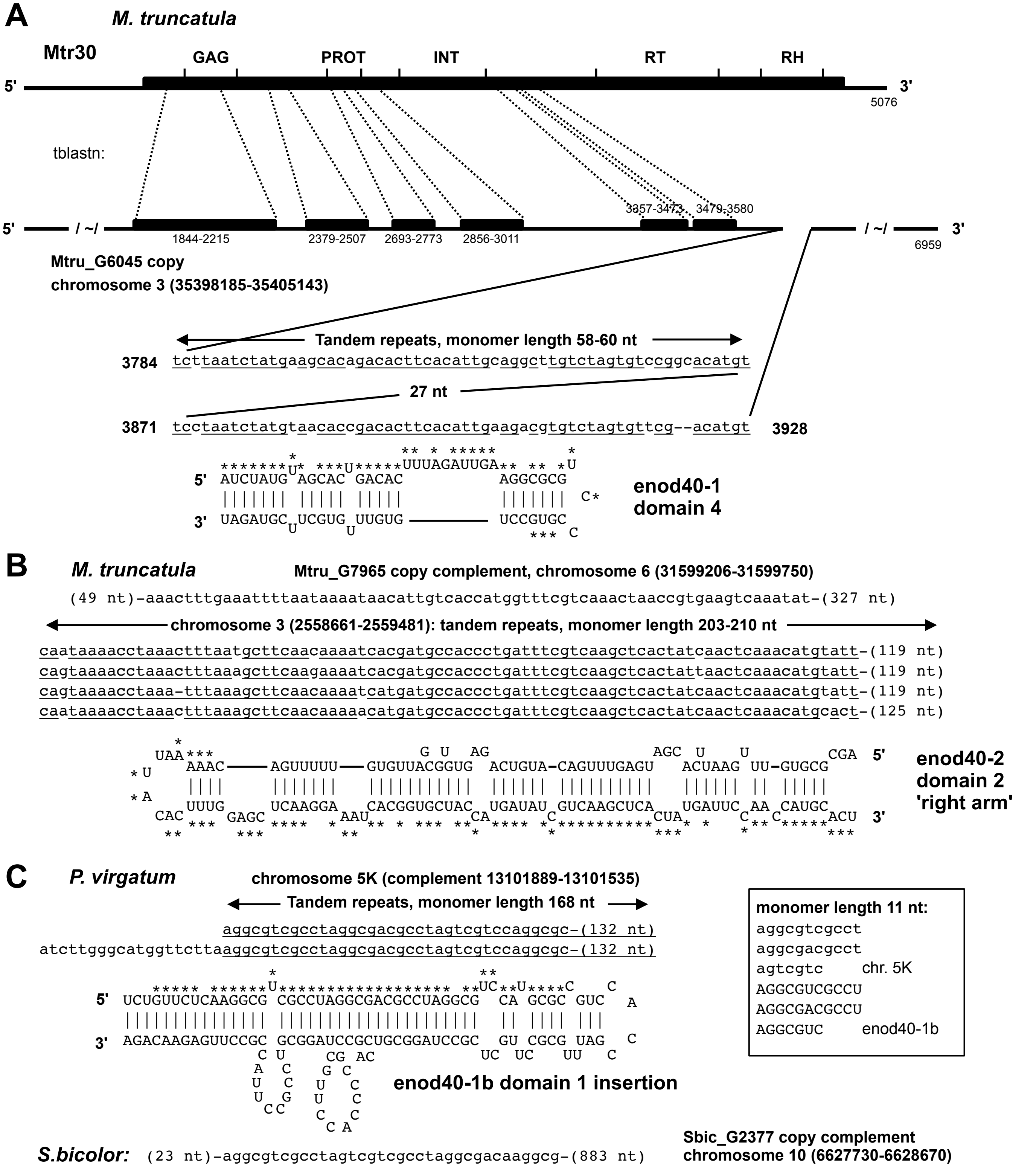

Supplementary Figure S2. Similarities between enod40 domains and repetitive DNA in *Medicago truncatula* and *Panicum virgatum*.
Accessions: *M.truncatula* chromosome 3, NC_053044.1; chromosome 6, NC_053047.1; *P.virgatum* chromosome 5K, NC_053140.1; *Sorghum bicolor* chromosome 10, NC_012879.2. Repetitive elements from the RepetDB database (Amselem et al., 2019): "Mtru_TEdenovoGr-B-G6045-Map3", Mtru_G6045; Mtru_TEdenovoGr-B-G7965-Map3, Mtru_G7965; "Sbic_TEdenovoGr-B-G2377-Map4", Sbic_G2377. Mtr30 is a LTR retrotransposon of the Ty1/Copia type ((Wang and Liu, 2008). The traces of transposon polypeptide - coding sequence in the Mtru_6045, identified by tblastn, are shown by black bars. Similar nucleotides in tandem repeats are underlined, asterisks denote similarities between enod40 RNAs and tandem repeat monomers.

A *L. japonicus* **Tandem repeats (TR): contig Lj3.0_chr0_16917 (BABK02016917.1, complement 11191-10910)**

Tandem repeats, monomer length 141 nt

```
TR   1 ctattatgtaatcgggttgattcggtcgcaactgcaattgcaatgctgatgcgggga  57
TR 142 ctattatgtaatcgggttgattcggtcgcaactgcaattgcaatgctgatgcgggga 198

TR  58 ctccaaaatccgttatgttatggccgcaattgcgattacggaccgcaatttgaaaccctа-ttagtacgtattaatttgacacat 141
TR 199 ctccaaaatccgttatgttatggccgcaattgcggttacggaccgcaatttgaaaccctа-ttagtacgtattaatttgacacat 282
       ** *****  *********  ** **** **** ***  *** *************** * ******
    34 CUUCAAAACUCGUUAUGUUGCGGUCGCACUUGCAGUUGUGGAUCGCAAUUUGAAACCCGAGUUAGUA 100 enod40-2 domain 2
             ((((((((.(((((((((.(((........))))))))))))).))).....))))))        "left arm" structure
```

B *N. schottii* **Tandem repeats (TR): scaffold7242_cov114 (QANU01065388, 13178-13446)**

Tandem repeats, monomer length 128/129 nt

```
TR             1 gcatgtctgattttaagatatgaatccccacaattgattct-gaagc-attggaagtgaaaaattt  64
TR 129 aactaactattggcttgtttagtttttagataaaaataactagaattgattat-gaggc-tctaaaagtgagaaaatt 204
                 ****** ******* *  **  **  * ** ********** * *** *** **** * *******
               1 GCAUGUUUGAUUUUCAUGUAGAAACACUCAGAAUUGAUUCUGGCAGCUAUUAGAAG-GGAAAAUUU  65 enod40-2 domain 4
                 ((((((((.((((.((((.((((((((.(((((((((.(((.((.((((((((-((.......     structure

TR  65 tcacttctagtgactattagaatcaaatgtaggtg-ttcccacttgaaaatcgaacatatactta 128
TR 205 tcacctccaataaccgtcattatcaatttagttgcttttaccttaaaatcaaacattcactta 269
       *********** ***** * ****** * ****** ** **** * ****
    66 UCACUUCUAGUAACUAUCAAAAUCAAUUCUAGGUG-UUUCCACAUAAAAAAAAAACCAAACAUGC 129 enod40-2 domain 4
       )).))))))))).)).))).))))))))).))))-)))).)))).....)))).)))))))))     structure
```

C *L. angustifolius* **Tandem repeats (TR): chromosome LG06 (NC_032014.1, complement 31318851-31318600)**

Tandem repeats, monomer length 125/127 nt

```
TR   1 gaatccgtctgcgagttgagtttaaaatgagaaagaagagaaatggaagagttcagatttatctcataagtg  72
TR 126 gagtctgtttgcgagttaagttttggatgggaaggaatagaaaaggaagacttaagagtcacctgataagtg 197
             ****************** * ** ** *** ** ** **** **** * ***** ** **** **
           1 GUUUGCGAGUUAAGUUUUGAAAGGAAAUGAAGAGUAAUGGAAAACUUCAUAGUCAUCUUAUAA-UG  65 enod40-3 domain 4
             ((((((((((..((((((((.(((((.((((((........((((...((((((...(((.-..     structure

TR  73 tgttttatggcttccaag---------tttttctttccctctatta--ctcgcaaacaaacctt 125
TR 198 tatttcatggttctgaac---------cctttcccttccaaaaccaaactcacaaactaaccct 252
       ****** **  *** **          ****** ******** ******   ****
    66 UAUUUCGUGACUCUAAAGUCUUCUCUUUCUUUCCAUUCCAAAAUCAAACUUGUAAAC  122 enod40-3 domain 4
       )))...))))))...))))...)))))).))))).)).))))))..)))))))))))       structure
```

D *C. arietinum* **Tandem repeats (TR): chromosome Ca1 (NC_021160.1, complement 29060736-29056241)**

Tandem repeats, monomer length 2248/2247 nt

```
TR    1 (791 nt)-cgaaaagttgacgaatctaaacttaattttaactaaatacatc-gatatttaa-(1404 nt) 2248
TR 2249 (792 nt)-cgaaaagttgacgaatctaaacttaattttaactaaatacatc-gatatttaa-(1403 nt) 4496
                   ********** * **** * *** ** *  ************* * * *****
               39 UGAAAAGUUGAUGUAUCUGAUCUUGAUCUAAAACUAAAUACAUCAGCUUUUUAA 92 enod40-2 domain 4
                  ((((((((((((((..(((....)))........))))))))))))))).  apical part structure
```

Supplementary Figure S3. Similarities between enod40 domains and tandem repeats in *Lotus japonicus*, *Nissolia schottii*, *Lupinus angustifolius* and *Cicer arietinum*.
The secondary structures of enod40 RNA (sub)domains are shown with bracket view. Asterisks denote similarities between enod40 RNAs and tandem repeat monomers.

A

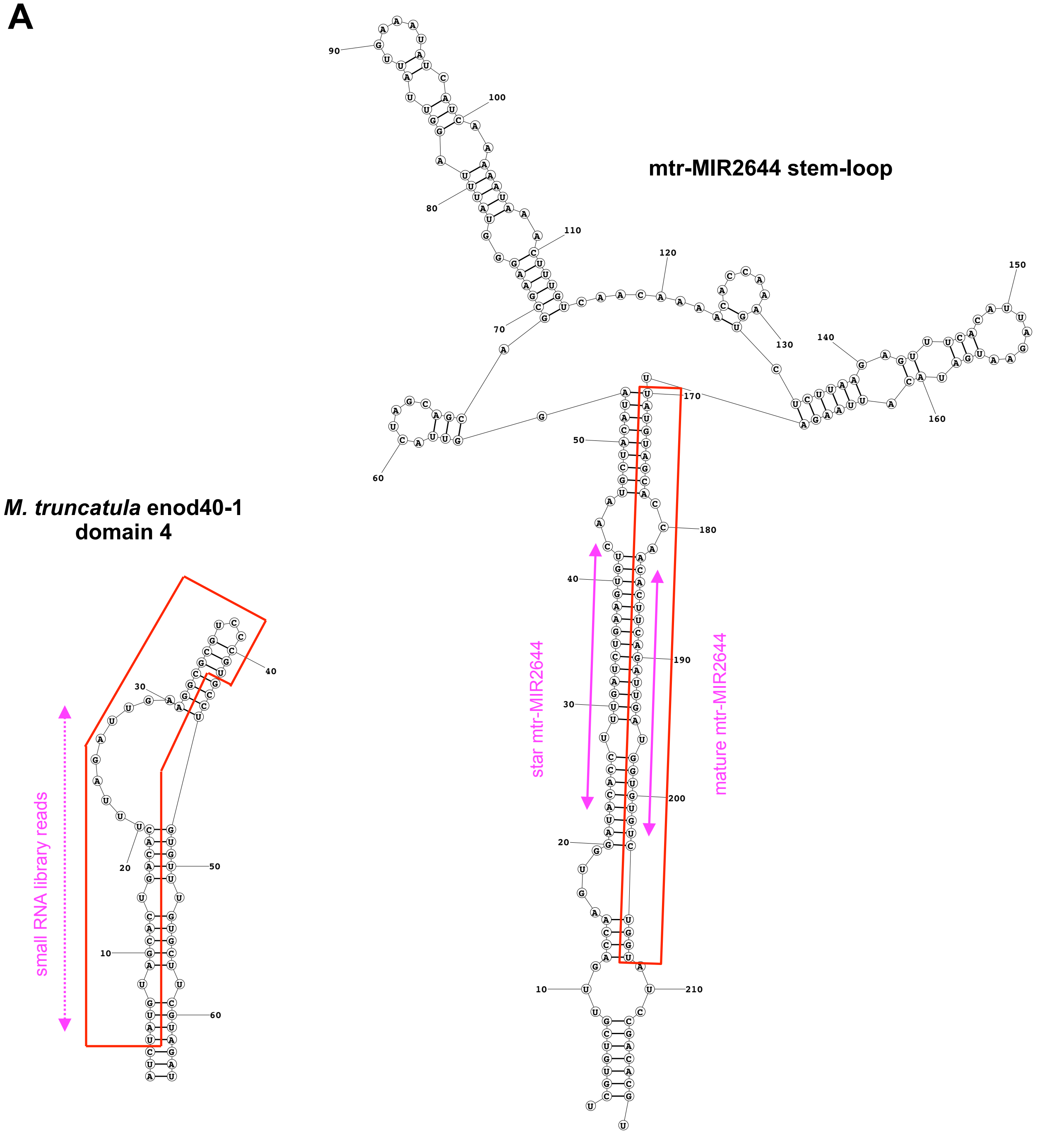

B

```
BLAST: E = 4e-04 (miRBase); E = 0.095 (M.truncatula genome)

  4 uauguagcacugacacuuuagauugaaggcgcguccccgu 42  enod40-1 domain 4
    ||||||||||  |||||| ||||||| || | |||  ||
170 uauguagcaccaacacuucagauugauggugugucuggu 208 mtr-MIR2644
```

Supplementary Figure S4. Similarity between the *Medicago truncatula* enod40-1 domain 4 and the mtr-MIR2644 microRNA. (A) Sequences yielded by BLAST alignment of the enod40 domain vs. the mtr-MIR2644 are boxed in the structures. (B) Blast alignment. The microRNA stem-loop structure and mature microRNA sequence are given according to the miRBase (https://mirbase.org) and PmiREN (https://www.pmiren.com), star microRNA according to the PmiREN data. The region in the *M.truncatula* enod40-1 domain 4 to which multiple small RNA library reads are mapped, e.g. from the sequence read archive with the SRA accession SRX651005, is indicated with dashed arrow.

A

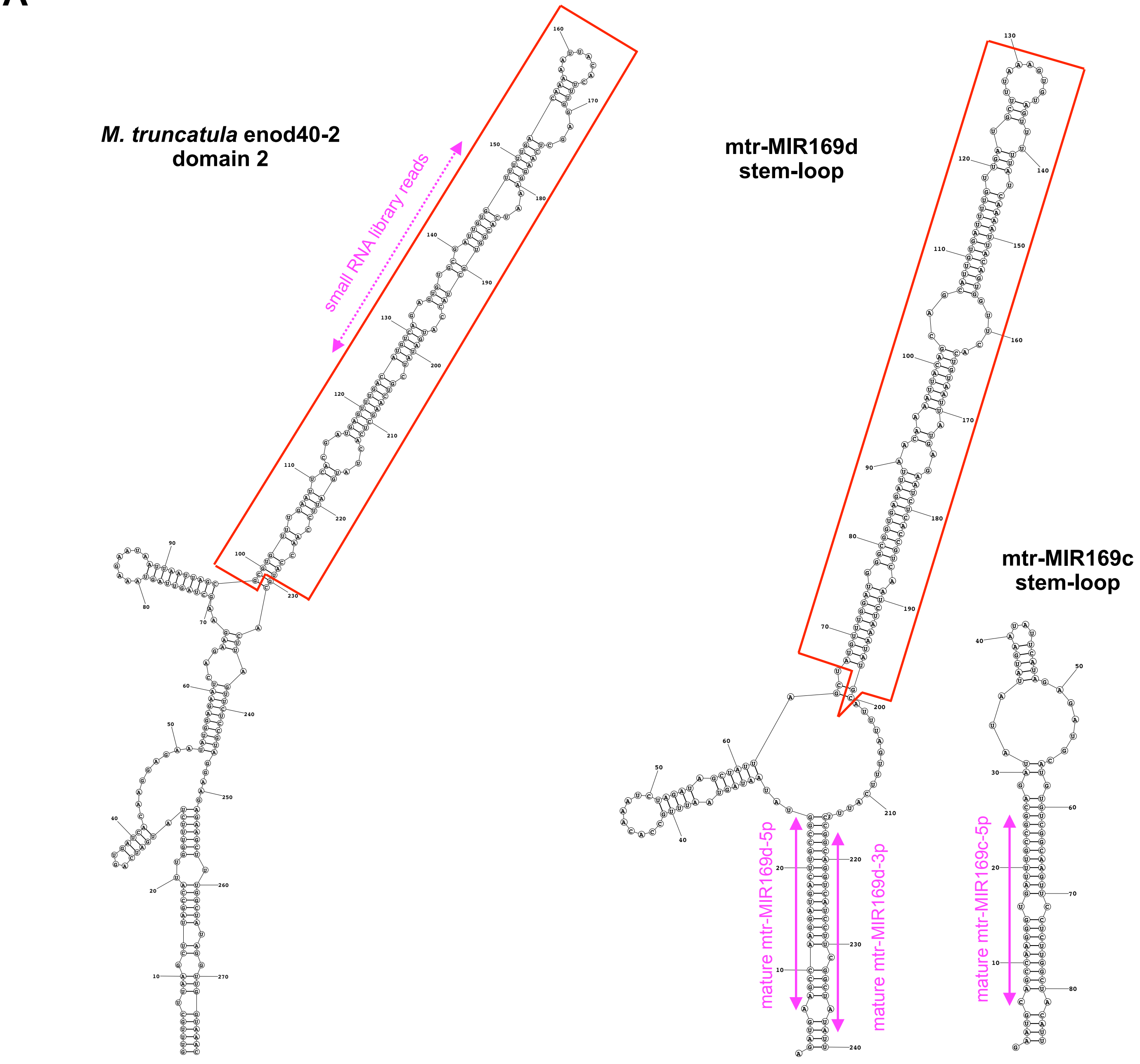

B

```
BLAST: E = 3e-11 (miRBase); E = 0.001 (M. truncatula genome)

 98 gcguguuugaauucacgaugaguuugacaug-ucagagugugcgauuguguuuuugacaaaaauuac 163 enod40-2 domain 2
    || | |||  ||| ||| |||| ||  |||  | | ||||  | | |||  |||||| ||||| |||
200 gcauauuuagauugacggugagauucucauaauuacagugaaccacuguaauuuugauaaaaacuac 134 mtr-MIR169d (complement)

164 acuuuggagc-ucaaggaaaucacggugcuaccaugauaucgucaagcucacuaugauuccaaccau 229 enod40-2 domain 2
    |||||  ||| ||||  |||||||  |||| |  | || | || || |||||      ||||| |||
133 acuuuaaagcaucaacaaaaucacaaugcugcuguaauuuuguuaaucucaccgcccauccaaacau  67 mtr-MIR169d (complement)
```

Supplementary Figure S5. Similarity between the *Medicago truncatula* enod40-2 domain 2 and the mtr-MIR169d microRNA. The related mtr-MIR169c stem-loop is shown in order to illustrate that the mtr-MIR169d contains the insertion similar to the one in the enod40-2 (see Figure 6). The microRNA stem-loop structures and mature microRNA sequences are given according to the miRBase (https://mirbase.org). Notations are similar to the Supplementary Figure S4.

A

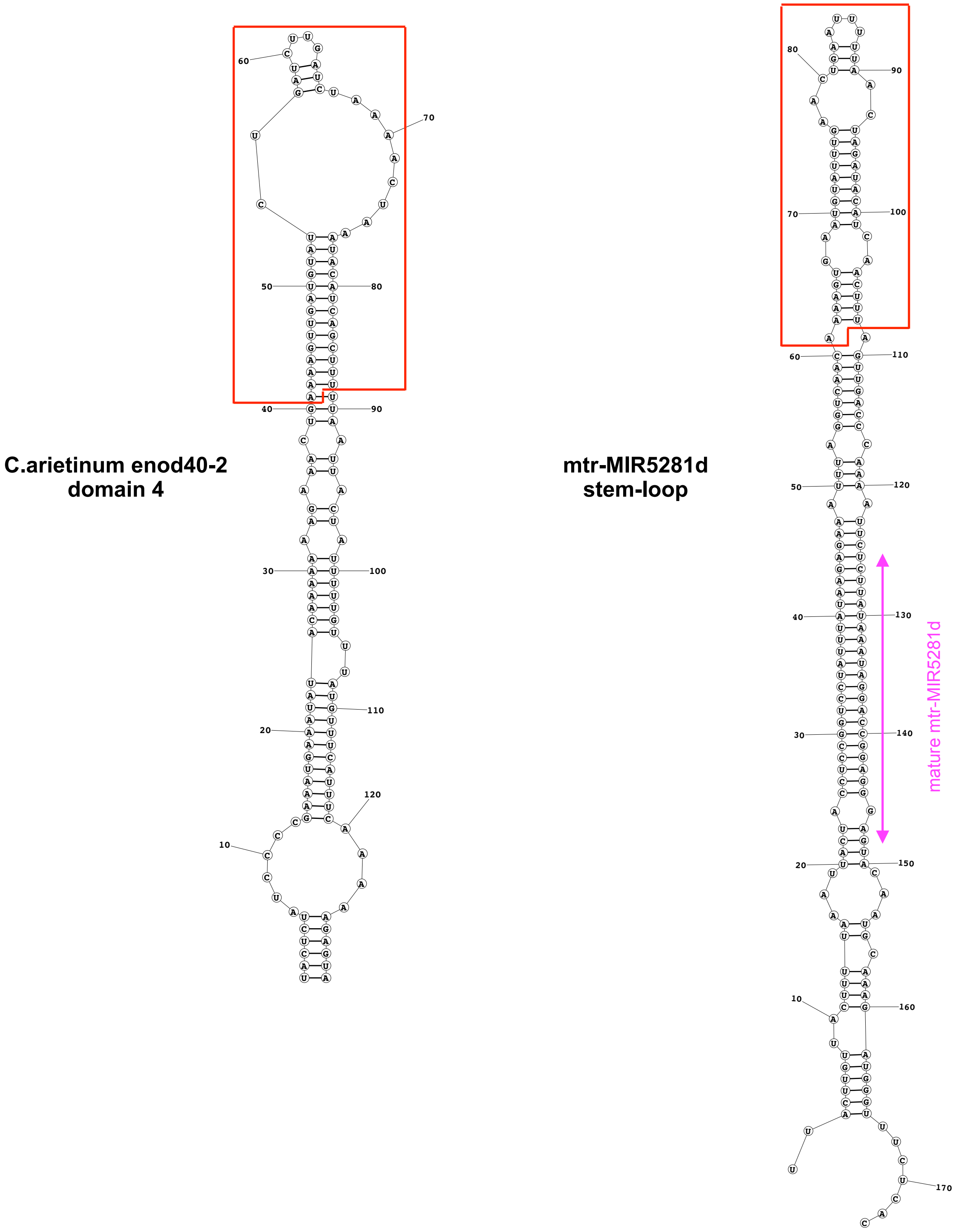

B

```
BLAST: E = 5e-05 (miRBase); E = 0.25 (M. truncatula genome)

41 aaaaguugauguaucugaucuugaucuaaaacuaaauacaucagcuuu  88 C. arietinum enod40-2 domain 4
   ||||||  |||||| ||| ||   || |   ||||| |||||||| ||||
61 aaaagugaauguauuugaacugaauuuuuaacuagauacaucaacuuu 108 mtr-MIR5281d
```

Supplementary Figure S6. Similarity between the *Cicer arietinum* enod40-1 domain 4 and the mtr-MIR5281d microRNA. The microRNA stem-loop structure and mature microRNA sequence are given according to the miRBase (https://mirbase.org). Notations are similar to the Supplementary Figure S4.

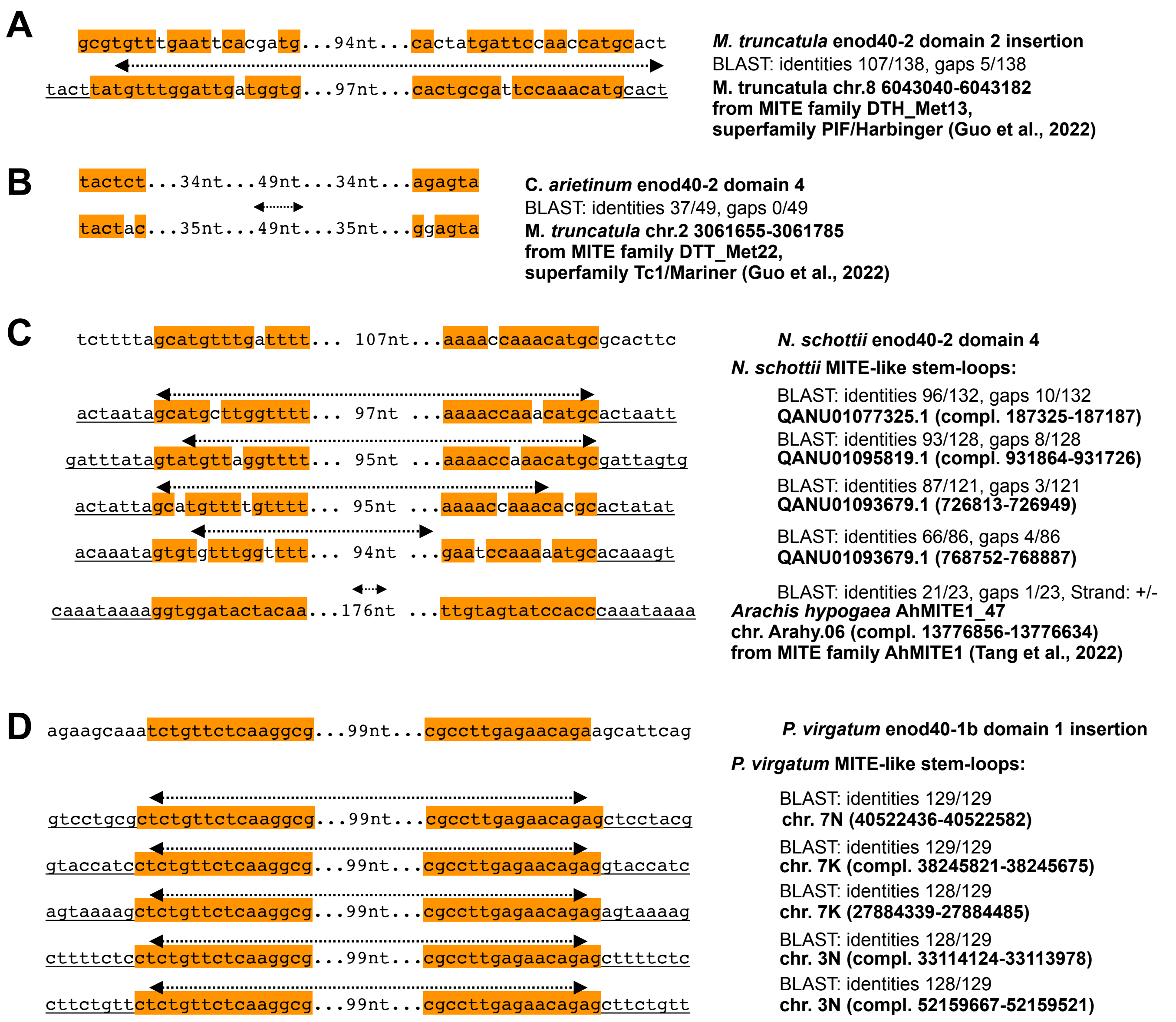

Supplementary Figure S7. Similarities between the enod40 domain insertions and MITE copies. Paired nucleotides at the stem-loop termini are labeled in orange. Target site duplication (TSD) sequences are underlined. Arrows indicate the regions covered by BLAST alignments. Chromosome (chr.) accessions: *M. truncatula* chromosome 2, NC_053043.1; chromosome 8, NC_053049.1; *A. hypogaea* chromosome Arahy.06, NC_037623.1; *P. virgatum* chromosome 3N, NC_053147.1; chromosome 7K, NC_053142.1; chromosome 7N, NC_053151.1. Compl., complementary sequence.
